# Using GIS to simulate tsunami evacuation guidance signs for the hearing impaired

**DOI:** 10.1371/journal.pone.0217512

**Published:** 2019-06-06

**Authors:** Ryo Horiike, Hisao Nakai, Tomoya Itatani, Fumie Shirai, Kaoru Konishi

**Affiliations:** 1 Medical Policy Section, Health Policy Department, Kochi, Kochi Prefecture, Japan; 2 Nursing Department, Kanazawa Medical University, Kanazawa, Ishikawa Prefecture, Japan; 3 Division of Health Sciences, Doctoral Course of Graduate School of Medical Pharmaceutical and Health Sciences, Kanazawa University, Kanazawa, Ishikawa Prefecture, Japan; 4 Division of Health Sciences, Osaka University Graduate School of Medicine, Suita, Osaka Prefecture, Japan; Fukushima Medical University School of Medicine, JAPAN

## Abstract

The Nankai Trough in Japan has been identified as a geological feature that could cause extensive damage in the event of a major earthquake. In this study, we investigated the impact of effective guidance signs for hearing-impaired individuals requiring special care when escaping to a tsunami evacuation building (emergency evacuation location) using geographical information system (GIS) and viewshed analysis. We selected an area we considered would suffer severe damage following a major earthquake and tsunami; we identified difficulties in the provision of escape routes. Using GIS, we determined the time required for escape and tsunami arrival time if effective signs were installed; we undertook such analysis using the height data of buildings in the target area. With effectively installed guidance signs, the average required evacuation time was 36.88 minutes; without such signs (which is currently the case in the target area), the average time was 47.10 minutes: that would result in citizens getting caught by the tsunami. Installing effective guidance signs would allow hearing-impaired individuals to escape to an evacuation building before being hit by the tsunami.

## Introduction

**Fig 1:**
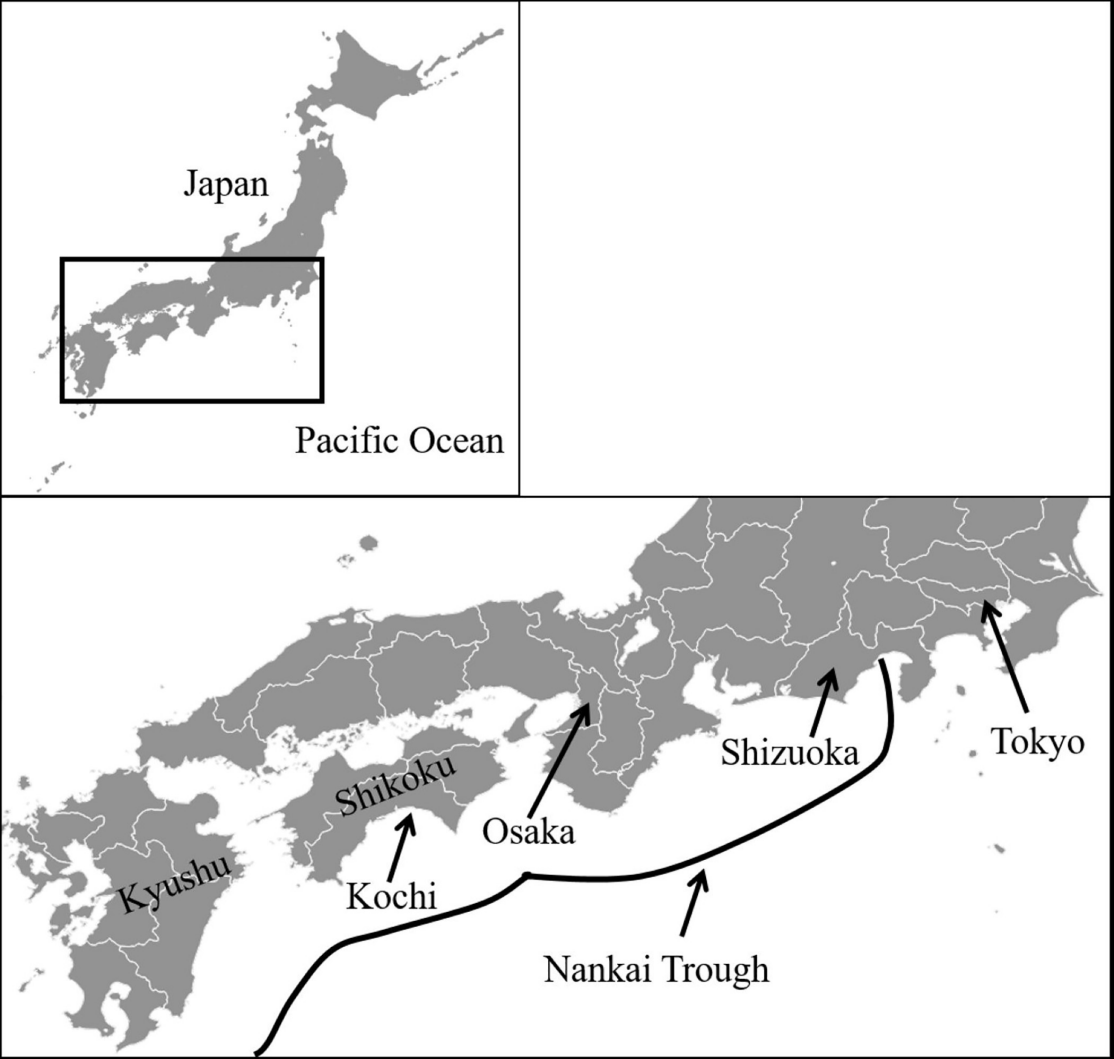
Location of the Nankai Trough.

In Japan, the probability of an earthquake occurring in the Nankai Trough (extending from offshore Shizuoka Prefecture to Shikoku and Kyushu islands) over the next 30 years has been estimated as 70%–80% (Fig 1) [1]. In the event of such an earthquake, the height of the subsequent tsunami in Kochi Prefecture (on the Pacific Ocean) has been calculated as 34 m, the highest in the whole country. Nationwide, the projected death toll would be49,000 people, of whom 37,000 (about 75%) would die as a result of the tsunami [2,3]. Japan is completely surrounded by sea, and so the tsunami could strike anywhere along its coastline.

It is against this backdrop that the Olympic Games will be held in Tokyo in 2020. Japan’s Cabinet Office and the Japan Tourism Agency have targeted foreign visitor numbers to the country in that year of 40 million, which is about twice the figure for 2015 [4]. Plans are in place to accommodate foreign visitors not only at Olympic venues but also in rural areas. Further, plans have been made to alleviate the burden for foreign visitors to Japan in the event of a disaster [4]. If a Nankai Trough earthquake occurred at that time, estimates indicate there would be up to 323,000 fatalities, including foreign visitors. Thus, efforts to reduce the destruction caused by such a tsunami are a matter of considerable urgency [1].

Guidelines and laws on disaster prevention measures clearly emphasize the necessity of provisions for people needing special care with respect to evacuation guidance. Such individuals include elderly people, the handicapped, infants, and foreign visitors [5]. Evacuation guidance for foreign visitors and people with disabilities states that various characteristics and circumstances, such as physical obstacles, have to be considered: because the target subjects may not be able to fully understand disaster information [6]. Moreover, it has been reported that individuals whose outward appearance does not suggest disability are at high risk in the event of evacuation [7]. Regarding measures for prompt and smooth evacuation, Article 9, Paragraph 1 of the Act on Promotion of Tsunami Countermeasures (concerning action in the event of a tsunami) addresses elderly people, the disabled, infants, visitors, and individuals who cannot understand Japanese. That section of the law stipulates the necessity to consider tsunami evacuation for at-risk individuals [8]: it underlines the need to provide evacuation guidance for such people.

Among individuals requiring special care, those with impaired hearing have information constraints [9]. Following the Great East Japan Earthquake in 2011, the mortality in affected areas among the total population was 1.03%; however, that figure among hearing-impaired people was 2.00%—approximately double [10]. Hearing-impaired individuals cannot understand disaster-prevention radio announcements; they cannot hear other people and do not understand information on TV without subtitles. Unless measures are taken to evacuate such subjects from a tsunami, they are at considerable risk [10].

One measure for dealing with a tsunami is rapid evacuation guidance. Tsunami evacuation signs are standardized using industrial criteria by the Ministry of Economy, Trade and Industry in the form of the Japanese Tsunami Evacuation Guide System (JIS Z 9097); they conform with Japanese Industrial Standards (JIS) [11]. Based on the concept of seamless design, JIS Z 9097 stipulates that tsunami evacuation guidance signs should be installed so as to direct unimpeded evacuation from a building in the event of a tsunami. JIS Z 9097 also states the desirability of indicating the name of the nearest tsunami evacuation refuge centre and the distance to it [11]. Signs for tsunami evacuation in buildings should appear on the first floor and rooftop; they should be designed so that they can be understandable from both short- and long-distance views.

It should be possible for evacuation to take place from a tsunami evacuation building (emergency evacuation place following a tsunami) at any time after the disadter; such buildings have to satisfy various requirements, e.g. in terms of height, robustness, and earthquake resistance [12]. Tsunami evacuation buildings assume the role of temporary emergency evacuation centres. However, after the Great East Japan Earthquake, there were cases of people remaining in a tsunami evacuation building for 24 hours [13]. If a hearing-impaired person (having limited information) sought shelter in a non-tsunami evacuation building, even though the building might be able to withstand the disaster, supplies and evacuation spaces would not be secure; thus, long-term stay could not be guaranteed. Without guidance signs for access to a tsunami evacuation building, a hearing-impaired person could get caught by the tsunami as they attempted escape to a non-tsunami evacuation building.

In Kochi Prefecture, which, as noted above, is projected to suffer more deaths than anywhere in Japan following a Nankai Trough earthquake, there is no effective evacuation guidance in conformity with JIS Z 9097. Municipal tsunami evacuation plans state only that guidance signs should be provided; there are no other details, such as regarding design and placement [14,15]. Previous studies investigating warning systems for tsunami evacuation have examined light detection and ranging (LiDAR) data, evacuation route-planning algorithms, evacuation system development based on geographical information system (GIS), and improving signs for such evacuation [16–23]. Many simulations have been conducted based on evacuation to safe areas and included such factors as the shortest routes, optimal direction of movement, and disaster prevention radio announcements [24–27]; however, those simulations have assumed that evacuees are actually aware of the shortest routes and optimal directions of movement and can receive such radio announcements in the event of an earthquake. Evacuees who lack such knowledge and awareness and require special also need to be considered in evacuation scenarios.

The purpose of this study was to clarify the effectiveness of tsunami evacuation guidance signs as a way for people needing special care to escape in the event of a tsunami. In this investigation, we examined the effect of unified guidance for evacuation to a tsunami evacuation building. We did so for hearing-impaired people requiring special care under the assumption of massive damage in Kochi Prefecture caused by a Nankai Trough earthquake. We conducted this research using GIS and viewshed analysis.

## Materials and methods

For our analysis, we collected data for June to October 2018.

### Target area and tsunami evacuation building

In Japan, the largest tsunami (34 m) would strike rural areas; evacuation in those areas is clear, involving such destinations as hills and mountains. In urban areas, however, the main focus for evacuation is buildings, and many buildings have the appropriate height to serve as tsunami evacuation buildings. Therefore, in the present study, we targeted urban areas. We selected an area we supposed would suffer severe damage following an earthquake and tsunami and that would present geographical difficulty with respect to furnishing escape routes. We set the following five parameters for our identified area: (1) location in the centre of Kochi city, which has about half the population of Kochi Prefecture [28]; (2) seismic intensity of 7, which is the highest predicted intensityfor a Nankai Trough earthquake [3] (3) estimated time for a 30-cm tsunami impeding human movement for 60 minutes [3]; (4) predicted tsunami flooding depth of 3 m, which would mean that the destruction of almost all wooden buildings in the area [3]; and (5) rivers to the east, west, north and south of the identified area as well as certain directions in which movement would be impossible in the event of an earthquake and tsunami.

We confirmed our target area on the Kochi Prefecture disaster prevention map [29]. There were two tsunami evacuation buildings on the southern side of the area [12]. A major river bounded the area’s north side, preventing evacuation in that direction. Thus, tsunami evacuation would have to proceed to the two buildings on the south side. For evacuation to the south side of that area, we therefore selected the building that could be reached first.

### Acquisition of building data

To determine the evacuation route and undertake viewshed analysis, we obtained data concerning the exterior of the identified building in the target area from the base map information download service of the Geographical Survey Institute (https://fgd.gsi.go.jp/download/menu.php) [30]. We used outer line data, which are 2-D building outlines that connect the surroundings of buildings with lines and express buildings as surfaces.

### Field survey and Google Earth

To determine the height of the targeted building, we counted the number of floors and obtained outline data from a field survey and used Google Earth. Then, following the method of the National Institute for Land and Infrastructure Management, we estimated the height of the building by multiplying the number of floors by the average floor height (3.5 m) [31,32]. In our field survey, we found a sign identifying the structure as a tsunami evacuation building [33]: the sign was placed at a height of 2.52 m. Other than that sign, there were no indications of the route to the tsunami evacuation building. In the target area, there were two non-tsunami evacuation buildings that had the same height as our identified building.

### Data input to GIS software

Using GIS software QGIS (version 2.18.3; QGIS Development Team; https://www.qgis.org/en/site/), we mapped the exterior of the building (including its height) and data relating to the tsunami evacuation sign [34].

### Tsunami evacuation guidance signs and visible area

For effective evacuation guidance for the hearing impaired, we set the distance between guidance signs as 20 m. Guidelines of the Fire and Disaster Management Agency put the effective range of evacuation exit light signs as up to 20 m [35]; guidelines of the Ministry of Land, Infrastructure, Transport and Tourism set the distance of guidance signs for individuals requiring assistance as up to 20 m [36]. Initially, we put guidance signs on buildings at the corners of street junctions. We used letters of the alphabet (A–H) to indicate the order of the signs, starting from the north. We established signs at eight junctions; we determined the latitude and longitude of each junction through geocoding. Next, between the signs installed at the junctions, we placed additional guidance signs; those additional signs were placed on buildings at a position that would allow unimpeded visibility over a distance of 20 m. The height of the additional guidance signs was the same as that of the sign on the identified tsunami evacuation building. We determined the 20-m intervals of the guidance signs using Visibility Analysis, which is a plug-in of QGIS [37]. After converting the output raster data to vector data, we obtained the area of the visibility.

### 3-D Model of tsunami and non-tsunami evacuation buildings

For the geographical relationship between the targeted tsunami evacuation building and non-tsunami evacuation buildings of the same height, we used Qgis2threejs, which is a QGIS plug-in. Qgis2threejs allows the use of 3-D computer graphics to reproduce the real-world situation, including building height data [38].

### Tsunami evacuation simulation

The evacuation route has to take into account the distance and time necessary for transit to the tsunami evacuation building; however, it is also necessary to consider the time loss due to transit to wrong buildings. We calculated those factors as follows. We set the evacuation speed at 1.66 km/h: we based that figure on the evacuation velocity of people requiring special care from the results of a survey of evacuees in the 2011 Great East Japan Earthquake [39]. It has been estimated that with a Nankai Trough earthquake, the tremors would continue for 3 minutes; thus, we set the duration of the earthquake as 3 minutes [3]. With the Great East Japan Earthquake, the average time to the beginning of evacuation after the tremor ceased was 22 minutes [39]. A survey following that earthquake identified the reasons for the delay as unawareness of an approaching tsunami, not hearing the alert, and the desire to first obtain information about the event [39] No previous studies have determined the time to reach a non-tsunami evacuation building, so we set that time as 2 minutes. In the target area, we estimated that a 30-cm tsunami (which would prevent evacuation) would arrive in 40 minutes and that the maximum of a 3-m tsunami would cause panic [29]. Thus, we set the available time to reach the tsunami evacuation building as up to 40 minutes.

## Results

**Fig 2:**
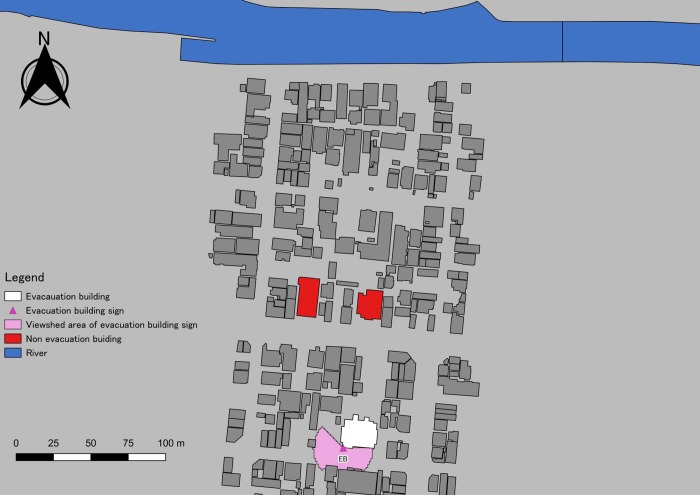
Area around our targeted tsunami evacuation building. We obtained the map included this figure from the Geospatial Information Authority of Japan, a Japanese national organization [30]. According to the terms of use, the copyright holder does not need to apply for permission to use it when it is used for academic articles, and it can be used freely and commercially by following the CC BY 4.0 license [30].

The area around our targeted tsunami evacuation building was 300 m long and 200 m wide. That area had two streets running north to south and four streets running east to west; they intersected at eight locations (Fig 2). The targeted building was situated on a street in the southern part of the area, and its height was 49 m. There were two buildings on a street north of the targeted building: they were both 45.5 m high, but they were not designated as tsunami evacuation structures. The evacuation sign in the area was at the south-western end of the tsunami evacuation building: it was the only sign identifying the building as such, and its height was 2.52 m. Without any other structures, the visible area with a 20-m radius centred on that sign was a maximum 1,256 m^2^; however, the visibility was obstructed, mainly by other buildings. Using viewshed analysis, we determined that the visible area centred on the tsunami evacuation building sign was 653.5 m^2^—just 52.1% of the maximum area.

**Fig 3:**
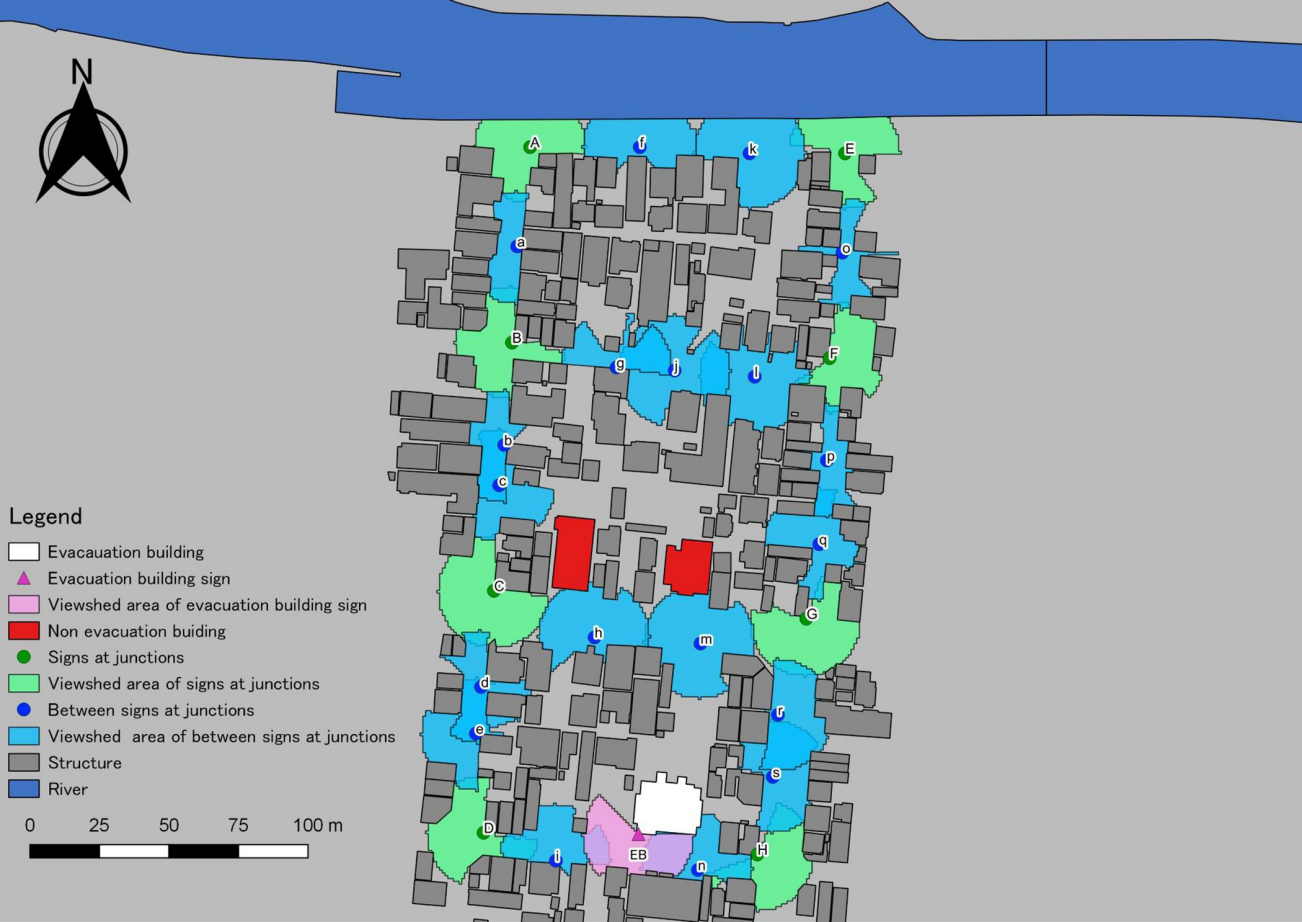
Visible area with tsunami evacuation guidance signs installed between the junctions. We obtained the map included this figure from the Geospatial Information Authority of Japan, a Japanese national organization [30]. According to the terms of use, the copyright holder does not need to apply for permission to use it when it is used for academic articles, and it can be used freely and commercially by following the CC BY 4.0 license [30].

With viewshed analysis and the signs installed at eight junctions, we found that the visible area with the signs increased to an average of 710.5 m^2^; that raised the proportion to the maximum area to an average of 56.6%. The distances between the signs on the junctions were 70 m or more; the visibility of the signs was not continuous. To effectively cover the route to the targeted evacuation building with appropriate visibility, we installed guidance signs between the junctions and conducted visible region analysis (Fig 3). The result was nine guidance signs between the installed junctions; one to three signs were required between the junctions. The visible range became 356.7 m^2^ (28.4%) to 993.0 m^2^ (79.1%); the difference was due to road width and structure (Table 1).

**Table 1:** Visible area of tsunami evacuation guidance signs installed between the junctions and proportion to the maximum visible area.

Signs	Symbol	Viewshed area (m^2^)	Proportion of maximum viewshed area (1256 m^2^; %)
**Evacuation building**	EB	654.1	52.1
**Signs at junctions**	A	648.2	51.6
	B	724.9	57.7
	C	952.1	75.8
	D	731.0	58.2
	E	614.8	48.9
	F	559.4	44.5
	G	817.4	65.1
	H	636.5	50.7
**Between signs at junctions**	a	390.9	31.1
	b	417.6	33.2
	c	584.8	46.6
	d	454.7	36.2
	e	597.3	47.6
	f	573.9	45.7
	g	437.2	34.8
	h	780.5	62.1
	i	549.7	43.8
	j	967.1	77.0
	k	870.6	69.3
	l	810.7	64.5
	m	993.0	79.1
	n	561.5	44.7
	o	360.0	28.7
	p	356.7	28.4
	q	558.1	44.4
	r	660.6	52.6
	s	699.2	55.7

**Fig 4:**
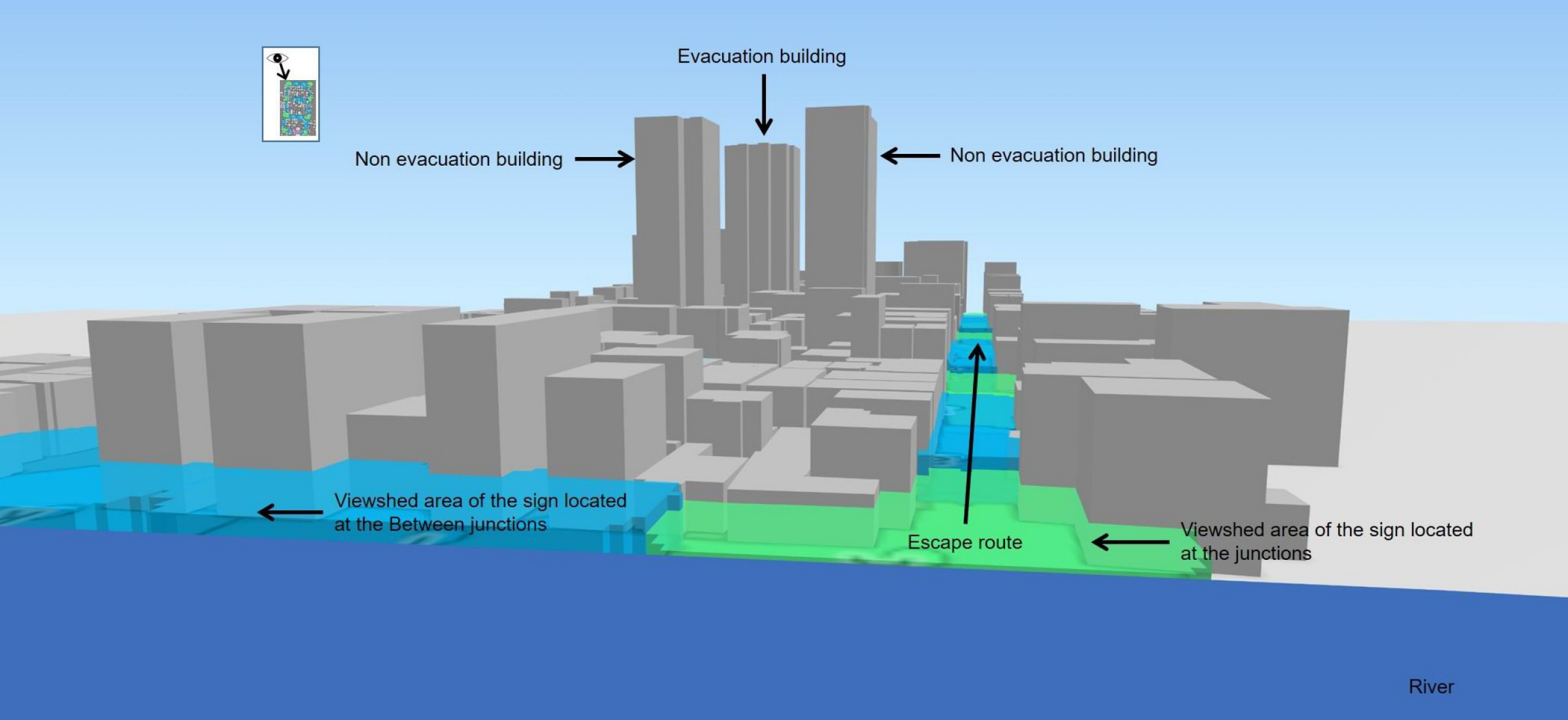
Appearance of tsunami and non-tsunami evacuation buildings. We obtained the map included this figure from the Geospatial Information Authority of Japan, a Japanese national organization [30]. According to the terms of use, the copyright holder does not need to apply for permission to use it when it is used for academic articles, and it can be used freely and commercially by following the CC BY 4.0 license [30].

To confirm the positional relationship between the target tsunami evacuation building and non-tsunami evacuation buildings, we created a 3-D computer graphic (Fig 4). The tsunami evacuation building was 3.5 m higher than the non-evacuation buildings; however, that difference was not externally obvious, and the non-tsunami evacuation buildings did in fact appear to be higher than the actual target building. There was no sign identifying the tsunami evacuation building as such in the upper part of the structure: the only indication of its status was a sign at a height of 2.52 m. However, in the event of evacuation, the area beyond a 20-m radius of the building would be out of the visible range, so the sign would be difficult to see. No guidance signs were actually installed in the area, so it would be impossible to guide at-risk individuals to the tsunami evacuation building. As noted above, if guidance signs were installed between junctions, it would be possible to create an uninterrupted continuous visible area for people needing special care.

**Fig 5:**
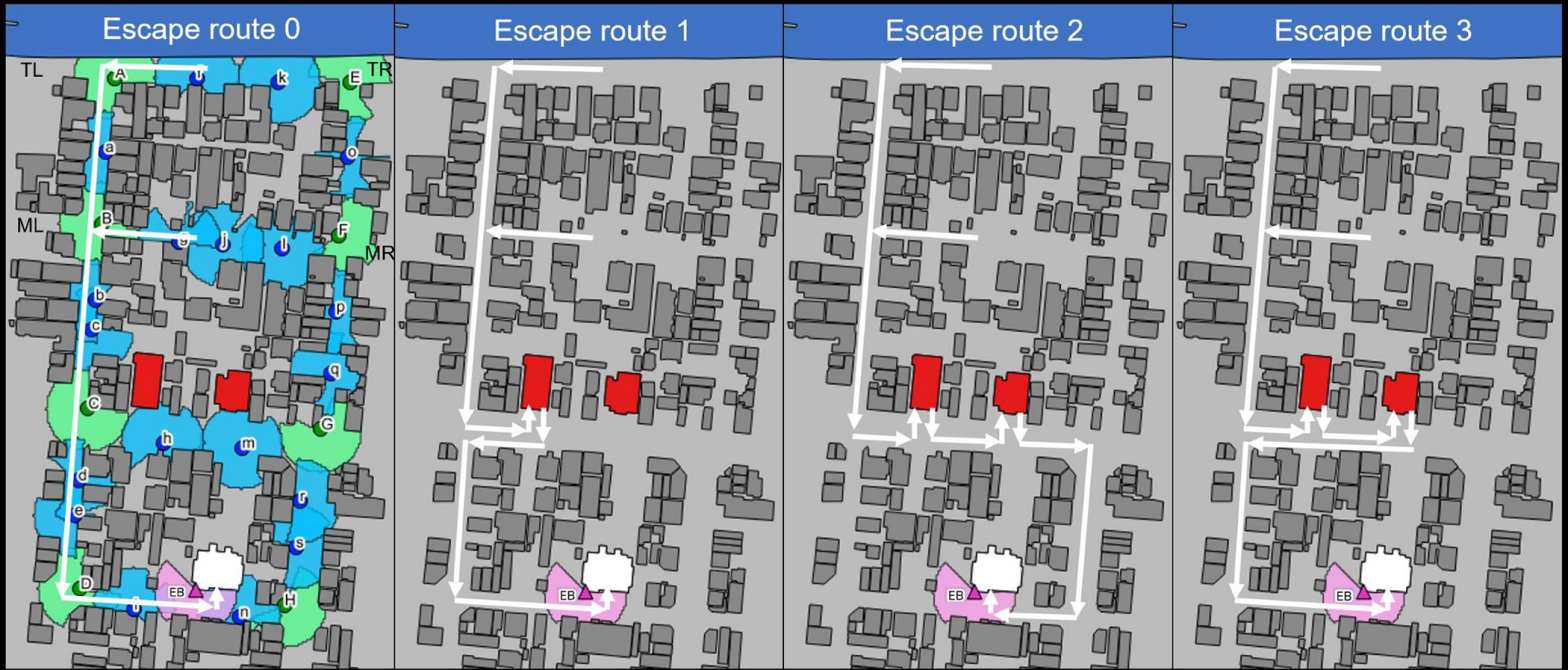
Possible escape routes to the tsunami evacuation building. White arrows indicate routes. For convenience of size, only the route on the left is displayed; however, the simulation was also conducted for the opposite side. TL (top and left): route to evacuate from the top to the left. TR (top and right): route to evacuate from the top to the right. ML (middle and left): route to evacuate from the middle to the left. MR (middle and right): route to evacuate from the middle to the right. Escape route 0: when tsunami evacuation guidance signs are installed without interruption. Escape route 1: escape via the nearest non-tsunami evacuation building; as in the real-world situation, signs are set up only for the tsunami evacuation building. Escape route 2: when returning to the original route by way of the two non-tsunami evacuation buildings; as in the real-world situation, signs are set up only for the tsunami evacuation building. Escape route 3: returning to the original route via the two non-tsunami evacuation buildings; as in the real-world situation, signs are set up only for the tsunami evacuation building. We obtained the map included this figure from the Geospatial Information Authority of Japan, a Japanese national organization [30]. According to the terms of use, the copyright holder does not need to apply for permission to use it when it is used for academic articles, and it can be used freely and commercially by following the CC BY 4.0 license [30].

The street with the tsunami evacuation building was in the south of the study area; the non-tsunami evacuation buildings were on a street to the north of the targeted structure. There were two other streets to the north of the non-tsunami evacuation buildings. Those two streets afforded the shortest route for evacuation south to the tsunami evacuation building. Since it could effectively cover the target area, we used the central point of the two streets to the north as the evacuation departure point (Fig 5).

We designated the streets located on the north side as the top (T) and the centre as the middle (M). We calculated the distance on the evacuation route to the left (L) and right (R) of the central part of each street, and we combined those data with information related to evacuation: we obtained the difference in the estimated tsunami inundation time of 40 minutes. We identified four evacuation route patterns (0 to 3) depending on the positional relationship between the tsunami and non-tsunami evacuation buildings and the positional relationship of the streets (Fig 5). We classified each route; for example, in the case of pattern 0, the route evacuated to the left from the top street was expressed as 0TL. In pattern 0, we installed continuous evacuation guidance signs in pattern 0 and established a route allowing directly escape to the tsunami evacuation building from each departure point without going through the non-tsunami evacuation buildings. Patterns 1–3 are without guidance signs, which is the real-world situation. After the departure point, pattern 1 branches to the left and right; it then passes through the closest non-tsunami evacuation building before continuing. After the departure point, pattern 2 has branches to the left and right; it then goes to the tsunami evacuation building without returning to the original course via the two non-tsunami evacuation buildings. After the departure point, pattern 3 branches to the left and right; it then returns to the original course via the two non-tsunami evacuation buildings.

We determined the required distance and time for each assumed evacuation route as follows (Table 2). Pattern 0 does not pass through a non-tsunami building and has the shortest evacuation distance: the average distance was 327.6 m, and the calculated time required for evacuation was 36.88 minutes. It was thus possible to reach the tsunami evacuation building within 40 minutes, which is the predicted tsunami inundation time. However, if evacuation began at the top street, there was a delay of about 2 minutes; that required a walking speed of 1.66 km/h, which is the speed of people requiring special care.

**Table 2:** Results of evacuation route simulation.

Evacuation pattern	Earthquake duration (min)	Evacuation start time after earthquake occurrence (min)	Evacuation distance (m)	Evacuation speed (1,660 m/h)	Transit time for evacuation (min)	Time for transit via non-tsunami evacuation building (min)	Number of transits via non-tsunami evacuation building	Time required for evacuation (min)	Predicted tsunami inundation time (min)	Time obtained by subtracting evacuation time from predicted tsunami inundation time (min)
0TL	3	22	374.6	1,660	13.6	0	0	38.60	40	1.40
0ML	3	22	304.2	1,660	11.0	0	0	36.00	40	4.00
0TR	3	22	352.6	1,660	12.8	0	0	37.80	40	2.20
0MR	3	22	279.1	1,660	10.1	0	0	35.10	40	4.90
1TL	3	22	434.8	1,660	15.8	2	1	42.80	40	-2.80
1ML	3	22	364.4	1,660	13.2	2	1	40.20	40	-0.20
1TR	3	22	455.6	1,660	16.5	2	1	43.50	40	-3.50
1MR	3	22	382.1	1,660	13.9	2	1	40.90	40	-0.90
2TL	3	22	475.4	1,660	17.2	2	2	46.20	40	-6.20
2ML	3	22	405.0	1,660	14.7	2	2	43.70	40	-3.70
2TR	3	22	514.4	1,660	18.6	2	2	47.60	40	-7.60
2MR	3	22	440.9	1,660	16.0	2	2	45.00	40	-5.00
3TL	3	22	527.0	1,660	19.1	2	2	48.10	40	-8.10
3ML	3	22	456.6	1,660	16.6	2	2	45.60	40	-5.60
3TR	3	22	543.8	1,660	19.7	2	2	48.70	40	-8.70
3MR	3	22	470.3	1,660	17.0	2	2	46.00	40	-6.00

Pattern 1 involves transit via the non-tsunami evacuation building closest to the evacuation route. The average distance required for evacuation was 409.2 m; the time required for evacuation was 41.85 minutes on average, which was about 5 minutes longer than with pattern 0. Thus, it exceeded the estimated tsunami inundation time. Even though the route was in the direction of the tsunami evacuation building, it would lead to time wasted at a building of similar height. Thus, it would create difficulties for a person requiring special care.

In pattern 2, the average distance required for evacuation was 458.9 m and average required time was 45.63 minutes. That is an increase of about 9 minutes compared with pattern 0 owing to the passage through two non-tsunami evacuation buildings.

For pattern 3, the average distance required for evacuation was 499.4 m and necessary time was 47.10 minutes. Those figures are the highest for the evacuation routes.

From the above patterns, it is clearly necessary to consider routes that allow direct access to the tsunami evacuation building. The visible area for guidance signs has to be appropriate for people requiring special care. Only pattern 0 permitted clear transit to the tsunami evacuation building.

## Discussion

In this study, we examined the effect of continuous guidance signs to tsunami evacuation buildings for hearing-impaired people. We found that with the current absence of such signs in the event of a tsunami, those individuals would have to escape via non-tsunami evacuation buildings. However, with a set of continuous signs, the visible region of guidance signs would extend to the tsunami evacuation building, allowing hearing-impaired people to escape. This study determined that in addition to helping individuals with hearing disabilities, seamless guidance signs would assist foreign visitors in recognizing the tsunami evacuation building from a radius of 20 m under any circumstances. One investigation found that to be effective in the event of a tsunami striking at night, signs for evacuation guidance and tsunami evacuation buildings should have a power-storing function through solar energy [11]. Solar-powered guidance signs are necessary owing to the power breakdowns and disruptions in communication networks that frequently accompany a major earthquake.

We analysed visibility in the target area study using data of buildings. However, in the real world, various objects, such as trees and cars, also obstruct the visible region. A more detailed analysis requires the use of LiDAR data; nevertheless, it is necessary to consider changes to the data content as a result of the season and human behaviour [17]. LiDAR is a remote-sensing method that generates 3-D information on the ground by illuminating a target with laser light from an aircraft [40]. In other countries, there is an active movement for access to necessary information with respect to evacuation simulation, such as LiDAR and building height data. However, in Japan, the open-data movement has not made conspicuous progress. For individuals requiring special care, it is vital to promote open data in Japan [41].

It has been reported that evacuation guidance signs do not make a cost-effective contribution to safety in the case of arterial roads [22]. The area covered in the present study did not have such thoroughfares; however, cost is an important factor when making policy decisions, and so it has to be taken into account.

With effective tsunami evacuation guidance signs, hearing-impaired people can escape independently to evacuation buildings. When a disaster strikes, citizens look for support from administrative organizations, including non-governmental organizations (NGOs), but it is necessary then to deal with many victims. Following the Great East Japan Earthquake at evacuation centres, in local communities, and for local authorities, it has been reported that individuals requiring special care often could not be properly identified [42]. It is necessary to provide assistance at evacuation centres for at-risk individuals, but many such people consider that they cannot hope to receive adequate help there [42]. Local authorities could present lists of at-risk candidates to support organizations, such as NGOs; however, owing to personal information protection, they are unable to do so. At general evacuation centres, it is necessary to ensure support for individuals with disabilities even in the great upheaval that accompanies a disaster. However, it is essential to ensure that the elements necessary for disabled people to act independently, such as guidance signs, are properly in place: that demands appropriate study and preparation. When introducing such elements, it is necessary to conduct training to confirm the effectiveness of installed tsunami guidance signs. One study has identified the necessity for at-risk individuals and their supporters to respond appropriately to evacuation preparations such that they can escape together with their supporters [43]. We infer from the results of our viewshed analysis that guidance signs should be provisionally installed and evacuation drills conducted; that would allow an accurate evaluation of evacuation methods. Nakai et al. performed a realistic evacuation simulation using surveys of home patients and their supporters in need of a power supply for health appliances, such as ventilators [44].

The present study was undertaken in line with efforts to identify disaster evacuation routes for hearing-impaired individuals and foreign visitors. That aim is 11th among Sustainable Development Goals of the United Nations: sustainable cities and communications. Toward reducing injuries in the event of a disaster, it is imperative to consider the needs of disabled and vulnerable people such that they have universal access to public spaces [45].

## Conclusions

We found in this study that the visible area of guidance signs was obstructed by nearby structures: sometimes, such obstruction reduced the visibility by about half. Thus, we undertook viewshed analysis using building data around the tsunami evacuation building. Following a tsunami disaster, visual information plays a vital role. The time required for victims to escape was delayed when they stopped at a non-tsunami evacuation building with the same height as the actual evacuation building: that would result in them being caught by the tsunami. It is necessary to install effective guidance signs for evacuation of the hearing impaired in the event of a tsunami.
